# Using a rapid assessment methodology to identify and address immediate needs among low-income households with children during COVID-19

**DOI:** 10.1371/journal.pone.0240009

**Published:** 2020-10-01

**Authors:** Shreela V. Sharma, Amier Haidar, Jacqueline Noyola, Jacqueline Tien, Melinda Rushing, Brittni M. Naylor, Ru-Jye Chuang, Christine Markham

**Affiliations:** 1 Department of Epidemiology, Human Genetics and Environmental Sciences, Michael & Susan Dell Center for Healthy Living, The University of Texas Health Science Center at Houston (UTHealth) School of Public Health, Houston, TX, United States of America; 2 Brighter Bites, Houston, TX, United States of America; 3 Department of Health Promotion and Behavioral Sciences, The University of Texas Health Science Center at Houston (UTHealth) School of Public Health, Houston, TX, United States of America; University of Texas Medical Branch at Galveston, UNITED STATES

## Abstract

**Objective:**

Brighter Bites is a school-based health promotion program that delivers fresh produce and nutrition education to low-income children and families. Due to COVID-19-related school closures, states were under “shelter in place” orders, and Brighter Bites administered a rapid assessment survey to identify social needs among their families. The purpose of this study is to demonstrate the methodology used to identify those with greatest social needs during this time (“high risk”), and to describe the response of Brighter Bites to these “high risk” families.

**Methods:**

The rapid assessment survey was collected in April 2020 across Houston, Dallas, Washington DC, and Southwest Florida. The survey consisted of items on disruption of employment status, financial hardship, food insecurity, perceived health status and sociodemographics. The open-ended question “Please share your greatest concern at this time, or any other thoughts you would like to share with us.” was asked at the end of each survey to triage “high risk” families. Responses were then used to articulate a response to meet the needs of these high risk families.

**Results:**

A total of 1048 families completed the COVID-19 rapid response survey, of which 71 families were triaged and classified as “high risk” (6.8% of survey respondents). During this time, 100% of the “high risk” participants reported being food insecure, 85% were concerned about their financial stability, 82% concerned about the availability of food, and 65% concerned about the affordability of food. A qualitative analysis of the high-risk group revealed four major themes: fear of contracting COVID19, disruption of employment status, financial hardship, and exacerbated food insecurity. In response, Brighter Bites pivoted, created, and deployed a framework to immediately address a variety of social needs among those in the “high risk” category. Administering a rapid response survey to identify the immediate needs of their families can help social service providers tailor their services to meet the needs of the most vulnerable.

## Introduction

On March 11, 2020, the World Health Organization declared the SARS COV-2 (COVID-19) a pandemic [1]. On March 13, 2020 the United States (U.S.) declared a national emergency concerning the COVID-19 outbreak, leading to numerous social distancing measures including, school closures, cancellation of public gatherings, and remote working [2]. Starting the week of March 16^th^ 2020, through the week of March 23^rd^ 2020, states began ordering schools to close for the academic year and issuing statewide stay-at-home orders, with many companies implementing work from home policies [3]. These social distancing measures had the detrimental consequences of disrupting food systems, businesses, and economies throughout the U.S. [4–6].

Rapid epidemiological assessment (REA) refers to post-disaster assessment methods that attempt to accurately assess a population by using the fewest resources in the shortest time [7]. These measures have included surveys, door-to-door assessments, surveillance methods, and screening and individual risk assessment using qualitative and quantitative methods [8]. Typically, during an outbreak investigation, these methods may be used to assess symptoms of disease to contain and prevent further outbreaks [7]. However, the application of a rapid assessment could extend to identifying social determinants of health needs during a disaster such as food insecurity, financial instability, unemployment, housing insecurity, and access to healthcare among vulnerable populations. This is particularly important to COVID-19, given that reducing risk of COVID-19 complications is related to maintaining optimal immune function and health, all of which are linked to these social determinants of health [4–6].

Brighter Bites is a non-profit, school-based health promotion program implemented across six cities (Houston, Dallas, Austin, New York City, Washington, D.C., and Southwest Florida areas) in the U.S., with the goal of delivering fresh produce and nutrition education to low-income children and families in underserved communities to mitigate food insecurity and improve dietary habits. Brighter Bites currently has an ongoing partnership with University of Texas Health Science Center at Houston (UTHealth) School of Public Health for research and evaluation. During the month of April 2020, amid the COVID-19 pandemic, while states were under “shelter-in-place” orders, Brighter Bites conducted a rapid assessment survey of families across four of the six cities (Houston, Dallas, Washington DC, and Florida). The survey screened families for food security, housing security, financial stability, employment status, transportation needs, access to childcare, and access to healthcare, to better understand the immediate needs of families, identify those with the greatest need, and provide them with critical resources during this time of crisis.

The purpose of this study is to demonstrate the methodology used to conduct the rapid assessment of needs using qualitative and quantitative measures among low-income households with children during the COVID-19 pandemic to identify those in greatest need, to present our findings, and describe the strategies taken to meet the participants’ needs.

## Methods

### Study sample

The rapid assessment survey was collected in April 2020 across Houston, Dallas, Washington DC, and Southwest Florida. The self-report survey was administered electronically using Formsite (Vroman Systems Inc., Illinois, USA) in English and Spanish to 16,500 BB families who were enrolled in the program in the 2019–2020 school year and had provided their contact information to the program. The participants in our study were predominately Hispanic (87%), while 7% were African American, and 5% were White/other. This is significantly higher than the sampled cities where the Hispanic population makes up at most 45% of the population in Houston, 42% in Dallas, 27% in Florida, and 11% in Washington D.C.

Completion of the survey was voluntary and informed consent was obtained from all participants prior to the start of the study. Data is collected by Brighter Bites non-profit organization, and then is de-identified and shared with the University of Texas Health Science Center (UTHealth) for analysis as part of a data sharing agreement and approved by the UTHealth Committee for Protection of Human Subjects. Data obtained in the first wave of survey responses, while cities were under “shelter-in-place” orders, were used for this paper.

### Data collection

The self-reported rapid assessment survey consisted of a 30-item questionnaire on COVID-19 related concerns, social determinants of health, and sociodemographics. The 5–10 minute in length survey was administered in English and Spanish.

Sociodemographic variables included child gender, respondents’ relationship to child, race/ethnicity of both parent and child, parent education level, parent employment status, and government assistance program enrollment. Program options included the Special Supplemental Nutrition Program for Women, Infants, and Children (WIC), Supplemental Nutrition Assistance Program (SNAP), Double Dollars, Medicaid, Medicare, National School Lunch and/or Breakfast Programs, and Children’s Health Insurance Program.

Household food security status was self-reported by the participants using the two-item Hunger Vital Sign™ screening questionnaire developed and validated by Hager et al. [9]. If the participant responded “often true” or “sometimes true” to either of the two questions, then the household was considered food insecure. If a participant answered “never true” to both questions, then the household was considered food secure.

Participants were asked about their concerns regarding various social determinants of health including financial stability, employment status, access to food, affordability of food, availability and affordability of housing, access to reliable transportation, access to childcare, access to clinic/doctor, or other concerns, which was open-ended for participants to fill in. Participants could check all that applied. Participants were asked to rate their health status. Responses included poor, fair, good, very good, and excellent.

STATA 15.0 (StataCorp College Station, TX) was used to perform all data analysis. Means, standard deviations, and frequency distributions were computed. Significance was set at p<0.05.

### Qualitative analysis

The open-ended question “Please share your greatest concern at this time, or any other thoughts you would like to share with us.” was asked at the end of each survey. The responses to this question were collated and analyzed for themes. Two trained UTHealth project staff conducted a thematic analysis to analyze the responses using an inductive approach, where codes and themes are derived based on the content from the survey data [10, 11]. The coding of the data was done initially through Microsoft Word using an open coding method by each coder. To ensure reliability of codes, coders collectively re-coded the data in Microsoft Excel. A codebook of codes and definitions was created, and codes were used to search for patterns and to identify possible themes.

Triaging for “high risk” families: “high risk” families were defined as the following: 1) If indicated in the open-ended question “Please share your greatest concern at this time, or any other thoughts you would like to share with us “the following response”: a) running out of food, b) diagnosed with COVID-19 and/or living with someone who has been diagnosed with COVID-19 and experiencing challenges, c) is ill and needs assistance, d) is about to lose their place of living, e) is about to lose their utilities, or f) no one at home is making an income; or 2) If indicated on the survey as “poor” on the question when they were asked to rate their current health status. If they met any of these categories, they were classified as “high risk”. Subsequently, these data were used to articulate a response to meet the needs of these high risk families.

## Results

The sociodemographic characteristics of the high-risk families are presented in Table 1. A total of 1048 families completed the COVID-19 rapid response survey, of which 28 families were triaged and classified as high risk (2.8% of survey respondents). Overall, the mean age of high-risk participants was 37 years, with 97% being female, 87% Hispanic, and 7% African American. A majority were Spanish speaking only (60%), 27% Spanish or English, and 10% only English. Interestingly, while 80% of children reportedly participated in the free/reduced meal program at the school, only, 30% reported receiving WIC, and 27% reported receiving SNAP assistance. Half of respondents were receiving Medicaid, 7% receiving Medicare, and 13% on CHIP. On average, there were five family members in the home.

**Table 1:** Sociodemographics, social needs, and health status of high-risk families (n = 71), Brighter Bites COVID-19 response survey.

	N	%	
**Cities**			
• Houston	54	76.06	
• Dallas	6	8.45	
• Washington DC	7	9.86	
• SW Florida	4	5.63	
**Does your family use the following?**			
• WIC	21	30.00	
• SNAP	19	27.14	
• Double Dollars	0	0.00	
• Medicaid/Texas Health Steps	35	50.00	
• Medicare	5	7.14	
• Free/Reduced meals	57	80.28	
• CHIP	9	12.86	
**Parent Race**			
• Black or African American	5	7.25	
• Mexican-American, Latino or Hispanic	60	86.96	
• White	0	0.00	
• Other	4	5.80	
**Child Race**			
• Black or African American	5	7.14	
• Mexican-American, Latino or Hispanic	59	84.29	
• White	2	2.86	
• Other	4	5.71	
**Home language**			
• Most or only English	7	9.86	
• Both English and Spanish equally	19	26.76	
• Most or only Spanish	42	59.15	
• Other language	3	4.23	
**Parent Gender**			
• Male	2	2.82	
• Female	69	97.18	
**Number of people live in your home**	**mean**	**SD**	
• Children	2.95	1.44	
• Elders	.14	.43	
• Adults	2.28	.79	
• Total	4.79	2.29	
**Parent age (years)**	36.6	7.24	
Health Status and Social needs			
		N	%
**Food insecurity due to coronavirus**			
• Food secure		0	0.00
• Food insecure		71	100.00
**Due to the coronavirus, are you concerned about any of the following in regards to you and your family? (check all that apply)**			
• Financial stability		60	84.51
• My employment status will change in the near future		32	45.07
• Availability of food		58	81.69
• Affordability of food		46	64.79
• Availability and/or affordability of housing		36	50.70
• Access to reliable transportation		8	11.27
• Access to child care		9	12.68
• Access to your clinic/doctor		25	35.21
**How would you rate your current health status?**			
• Poor		12	16.90
• Fair		23	32.39
• Good		20	28.17
• Very good		8	11.27
• Excellent		8	11.27

During this time frame, 100% of the high risk participants reported being food insecure, 85% were concerned about their financial stability, 82% concerned about the availability of food, and 65% concerned about the affordability of food. They were also concerned about the availability/affordability of housing (50%), while 45% were concerned their employment status would change in the near future. Other concerns were access to reliable transportation (11%), access to childcare (13%), and access to a clinic/doctor (35%). Almost half the participants (49%) reported being of fair or poor health status during this time.

While overall results of the qualitative thematic analysis for the n = 1048 participants are presented elsewhere (Sharma et al., under review), a qualitative analysis of participant responses in the high-risk group revealed four major themes presented in Table 2: fear of contracting COVID-19, disruption of employment status, financial hardship, and exacerbated food insecurity. Fear of contracting COVID-19 was an immediate threat for the families as is witnessed in one of the comments *“My daughter was diagnosed with coronavirus and I am very scared for all the members of my family”*. Moreover, loss of employment was of major concern leaving parents with little or no resources to meet their family’s needs. One parent said, *“I don’t have enough work*, *I’m a single mother*, *I have two children and I no longer have enough for food for my children and I’m behind with the rent*.*”*

**Table 2:** Themes from qualitative triage assessment of the high-risk families (n = 71), Brighter Bites COVID-19 response survey.

Themes	Example Comments	Response (what did Brighter Bites do)
**Fear of contracting COVID-19**	“My daughter was diagnosed with coronavirus and I am very scared for all the members of my family.”	{{e.g. BB produce vouchers provided, grocery drop off}} • Provided language-appropriate information and resources on safety measures–hand washing/sanitizer, wearing masks (talked about various items that could be a mask if they did not have access to any) social distancing and staying home. • Encouraged to seek testing if there was a concern of potential COVID-19 infection. • Provided information on community resources for clinics, doctors and testing sites by way of family resource links
	“We are worried that my husband does not have a job and we do not know how we will be able to buy food, pay the mortgage, electricity, water, the internet, what will happen to our future, we are very afraid, regarding the virus, of taking our children to the doctor, to the dentist, etc.”	
	“I don't want to go to the store and I no longer have food, afraid of going out.”	
**Disruption of employment status**	“I was left without work, and I don't have for supplies, or bills, or food.”	• Provided information on community resources for employment and unemployment benefit services. • Provided electronic $50 gift card to a local retail store. If they did not have access to an email, the $50 gift card was printed and hand-delivered to them.
	“I'm very worried because I haven't had work for three weeks and I don't have money to pay rent, electricity, or water. My work has been reduced by 90%, I'm an assistant housecleaner.”	
	“I don’t have enough work, I’m a single mother, I have two children and I no longer have enough for food for my children and I’m behind with the rent.”	
	“There's no work, and you still have to pay rent, and there's not enough money for food.”	
**Financial Hardship**	“My biggest worry is not being able to pay next month's rent and not knowing where to go.”	• Provided information on community resources for assistance programs, housing, transportation, child care and other services such as free or reduced internet access by way of family resource links • Cross-checked that they were receiving the produce vouchers to local grocery retail store and updated contact information accordingly. • Sent electronic $50 gift card to local retail store. Multiple electronic $50 gift cards were sent to families that communicated continued hardship. If they did not have access to an email, the $50 gift card was printed and hand-delivered to them.
	“Not having food for my children and frustration because I don't know what I'm going to do to pay rent. Thanks to you I have had some food. Thank you very much. May God give you more to continue helping. God bless you.”	
	“My main worry is that I do not have for the rent and food and we are two families who live here and neither of us are working and both families have children.”	
	“I have a water bill of $ 2141.79 and it will be cut off the 13th of this month. I'm in panic of being left without water.”	
	“I am worried about not being able to pay my rent next month (May_2020) because we are out of work.”	
**Exacerbated Food Insecurity**	“I don't have enough food for my children.”	• Provided dates and times for community food distributions and Brighter Bites food distributions. • Sent electronic $50 gift card to local retail store • If they did not have access to an email, the $50 gift card was printed and hand-delivered to them. • If they could not go shopping due to barriers such as lack of transportation, groceries were bought and delivered to them. (Family was asked to share food preferences) • Families received a $25 produce voucher to the local grocery retail store.
	“I am worried about how to feed the children because there is no work right now, I am worried about paying my debts”	
	“My biggest worry at the moment is food for my family.”	
	“I feel worried because I'm not giving enough vegetables and fruit to my children since my husband only works 3 days, and I'm not working because my baby was just born, so there are 4 children and 2 adults and I'm short of food and diapers for my baby, but what I need is food for the family. Thank you.”	

Loss of employment further led to financial instability leaving parents unable to pay their bills or buy food. One parent commented, “*My biggest worry is not being able to pay next month's rent and not knowing where to go*.” This financial uncertainty also left parents concerned about their food security, and about how they were going to access healthy foods with the limited produce available and the increasing prices in the grocery stores. One parent described their concerns by saying, *“I feel worried because I'm not giving enough vegetables and fruit to my children since my husband only works 3 days*, *and I'm not working because my baby was just born*, *so there are 4 children and 2 adults and I'm short of food and diapers for my baby*, *but what I need is food for the family*.*”*

**Fig 1:**
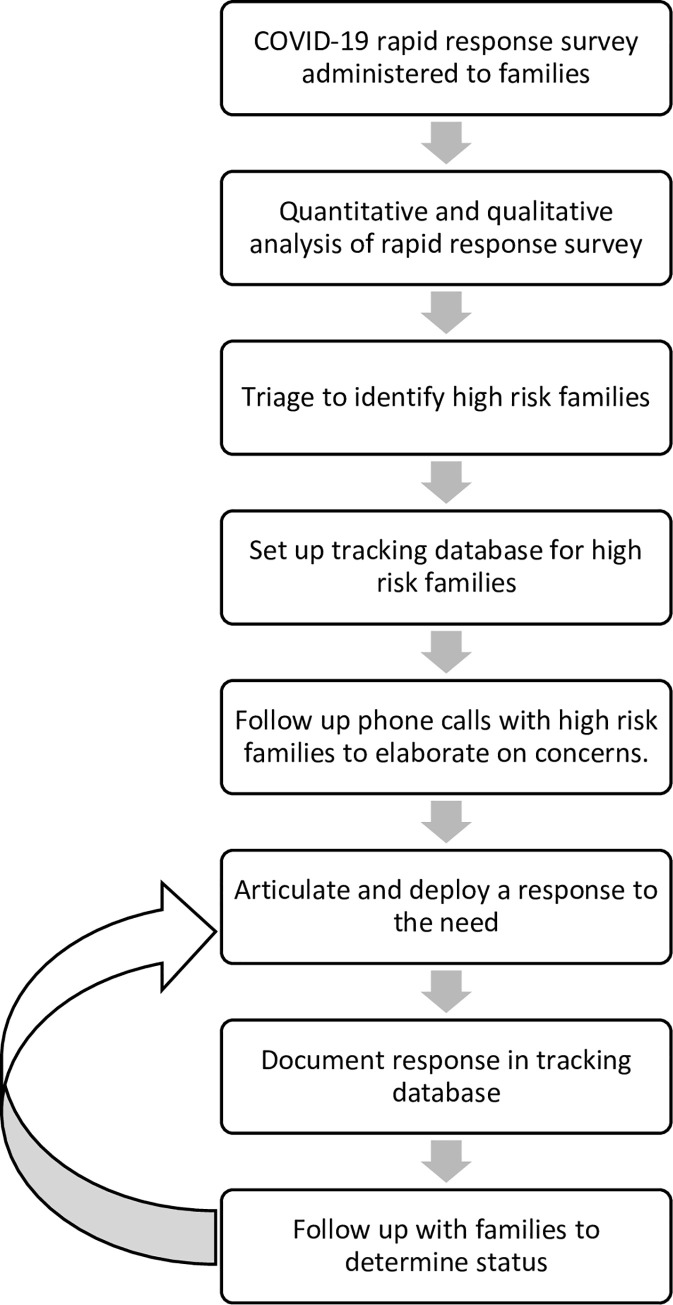
Flowchart of triage and response to need for high risk families, n = 71.

Brighter Bites response to high risk families: In response to these aforementioned needs, Brighter Bites pivoted and created an infrastructure and a standardized framework to meet the needs of these high-risk families (see Fig 1). A core, centralized group of Brighter Bites staff was assigned specifically to address these families’ immediate concerns. Following the triage, trained Brighter Bites staff made follow up phone calls to each high-risk family and obtain more details regarding their concerns and assistance needed. A tracking database was created, in which all phone calls and family concerns were tracked for each family. This information was then relayed back to the Brighter Bites leadership where a tailored response was generated for each family. Brighter Bites partnered with public health and medical schools’ faculty and students to develop educational materials and informational resources to provide to the families (Haidar et al., under review). Responses ranged from a) providing immediate financial relief in the form of gift cards to local retail stores, b) grocery drop off to families unable to leave home, and c) providing area-specific resources via text, email and phone regarding food distributions, financial assistance, safety from eviction due to inability to pay rent, COVID-19 testing sites near their homes, participation in government assistance programs etc. All responses were documented in the tracking database for each family. A 100% of the high-risk families were reached through this process.

## Discussion

The COVID-19 pandemic has had a major impact on the U.S. economy leading to a worldwide economic crisis [6]. The most vulnerable populations in our communities have been disproportionality affected by the direct and indirect health, social, and financial hardships of COVID-19 [12, 13]. Low-income, vulnerable populations contribute the most to healthcare costs, are more likely to suffer from health disparities, have increased risk factors, and have fewer resources for promoting health and treating illness [14]. It is therefore imperative that pertinent health, financial, and critical needs resources are provided in a timely matter to families and communities in need. This study provided insight into how organizations could potentially identify and respond to the needs of communities in times of crisis and demonstrates how to effectively reach families that are struggling and need support.

Social factors play a critical role in determining health outcomes [15, 16]. Addressing the social determinants of health of underserved communities through rapid response is important to reduce health disparities and improve health outcomes associated with COVID-19 and other comorbidities [12]. Minority populations, specifically African-American and Hispanic have been disproportionately affected by COVID-19, with increased risk of complications and mortality [17–19]. These same populations also suffer the most from chronic diseases such as obesity, diabetes, respiratory disorders, and other pre-disposing conditions, accounting for the majority of healthcare costs [20]. Often times, these diseases are also rooted in unequitable social and structural needs surrounding employment, transportation, housing, poverty, food, education, and health literacy [21, 22]. Conducting a COVID-19 rapid response survey was a purposeful decision on part of Brighter Bites to identify those with highest need during this time of crisis and develop a framework to immediately address a variety of social needs among those in the “high risk” category. Due to the rapid transmission of coronavirus, many cities including the four in our study were rapidly brought to a halt with many states issuing state-wide stay-at-home-orders, closing schools, restaurants, and businesses [3]. For low-income families this resulted in loss of wages and increased financial instability adding further strain to previous economic hardships. Administering a rapid response survey during such times is important to help identify the immediate needs of these families can help social service providers re-direct their services to meet the needs of this vulnerable population.

In response to the COVID-19 pandemic, Brighter Bites pivoted rapidly to invest its network into addressing urgent needs of their participating families. Brighter Bites used the expertise of public health faculty and students of their partner institutions to create and rapidly provide resources for their families. For example, if a parent indicated that they were fearful of contracting COVID-19, the team discussed safety measures such as handwashing, social distancing, and various items that could serve as a mask. If a parent was concerned about paying for rent or food, then Bright Bites team provided information on community resources for food, bill relief, and ensured they were receiving vouchers, and gift cards to local grocery stores. Details regarding these strategies and their dissemination is provided elsewhere (Haidar et al., under review). By having a triaging and tracking system, along with a centralized response team and strategic partners, in place, Brighter Bites was able to have a targeted response to meet the needs of 100% of families with the highest needs during this time. Social service organizations can use similar strategies to identify and address the needs of their populations rapidly during times of disaster.

This study has some limitations and strengths. The study sample is a sub-sample of families who participated in Brighter Bites in the 2019–2020 school year. The sample size varied across each city as family enrollment in the Brighter Bites program is not proportional to size of the city, and likely those families who needed the most help responded to the survey. Finally, survey was conducted to families electronically which introduces a selection bias as a limitation due to the fact that only families that had access electronically could participate. However, in this phase of the pandemic, given closures of schools and businesses, this was the most effective way to reach people. Finally, the qualitative themes observed in our study cannot be sorely attributed to the pandemic, and could be a result of other multitude of factors which remain to be seen. The purpose of this study was to identify the families with the highest need for social services during a time when all cities surveyed were in “shelter-in-place” and deploy an immediate response, which was successfully achieved. Finally, the timeline from the pandemic occurring to our response to these families is one of the major strengths of this paper.

## Conclusion

Our study provides the methodology and framework to screen at-risk low-income families for social needs during the time of the COVID-19 pandemic, and to provide a timely response to these critical needs. In the future, this framework could be used for other pandemics and times when an immediate response to screen at-risk families will be needed.

## Supporting information

S1 Data (XLSX)

S1 File (PDF)

S2 File (PDF)
