# Supplementary material for: Using a rapid assessment methodology to identify and address immediate needs among low-income households with children during COVID-19
Source: PLoS One. 2020 Oct 1;15(10):e0240009. doi: 10.1371/journal.pone.0240009 (PMC7529270; doi:10.1371/journal.pone.0240009)
Supplement: S1 File — (PDF) [file pone.0240009.s002.pdf]

COVID-19 Response Form / Formulario de Respuesta COVID-19

33% Complete

Dear Parent,

We are conducting a survey to understand how you are preparing and coping with the spreading COVID-19 (coronavirus), especially when it comes to your needs with food.

- Please answer to the best of your ability.
- There are no right or wrong answers.
- There is no risk for filling out the survey. By filling out the survey you agree to participate in the survey.

*NOTE: Red asterisk * means question is required.*

***Brighter Bites COVID-19 Response - Parent Survey***

**1. How much have you seen or heard about the COVID-19 (coronavirus)?** *

- ◯ A great deal
- ◯ A fair amount
- ◯ Not very much
- ◯ Nothing at all

**2. Due to the coronavirus, are you concerned about any of the following in regards to you and your family? (check all that apply)** *

- ☐ Financial stability
- ☐ My employment status will change in the near future
- ☐ Availability of food
- ☐ Affordability of food
- ☐ Availability and/or affordability of housing
- ☐ Access to reliable transportation
- ☐ Access to child care
- ☐ Access to your clinic/doctor
- ☐ Other

**3. DURING THE PAST 7 DAYS, HOW MANY TIMES DID YOUR FAMILY:** *

| | Never | 1-2 times per week | 3-4 times per week | 5-6 times per week | 7+ times per week |
|---|---|---|---|---|---|

| | | | | | |
|---|---|---|---|---|---|
| Eat food from any type of restaurant? This includes restaurants such as fast food, sit down restaurants, buffet restaurants, taco shops, donut shops, and pizza places. | ◯ | ◯ | ◯ | ◯ | ◯ |

**4. Due to coronavirus, has your frequency of eating food from restaurants changed?** *

◯ Increased

◯ Decreased

◯ Stayed the same

**5. How true do you find the following statement? Please mark one answer choice for each statement. Due to coronavirus:** *

| | Often True | Sometimes True | Never True |
|---|---|---|---|
| You worried whether your food would run out before you got money to buy more. | ◯ | ◯ | ◯ |
| The food you bought just didn't last and you didn't have money to get more. | ◯ | ◯ | ◯ |

**6. Due to coronavirus, currently how often do you buy or get fruits and vegetables and other groceries for the family from a large grocery store or super market? (such as Randall's, HEB, Kroger's, Fiesta, Whole Foods, Sprouts, Sam's club, Costco, Wal-mart, or Target) )** *

◯ Never

◯ Less than once a month

◯ 1-2 times per month

◯ 1 time per week

◯ 2+ times per week

**7. Due to coronavirus, currently how often do you buy or get fruits and vegetables and other groceries for the family from these locations?** *

| | Never | Less than once a month | 1-2 times per month | 1 time per week | 2+ times per week |
|---|---|---|---|---|---|
| A small local store or corner store (usually locally owned and do not sell gas), or a convenience store (such as 7-11 or mini market usually sell gas) | ◯ | ◯ | ◯ | ◯ | ◯ |
| A farmer's market/food co-op/farm stand | ◯ | ◯ | ◯ | ◯ | ◯ |
| A food bank/food pantry, or other food distributions | ◯ | ◯ | ◯ | ◯ | ◯ |

**8. At this time, how do you or your family member(s) shop at these grocery stores or super markets? (check all that apply)** *

☐ Physically shop inside the store

- ☐ Shop online and Curbside pick up
- ☐ Shop online and delivered to home

**9. Due to the coronavirus, has your consumption of fruits and vegetables:** *

- ○ Increased
- ○ Decreased
- ○ Stayed the same

**10. Which of the following is true for you? (According to CDC information, symptoms of coronavirus include: fever, cough, shortness of breath)** *

- ○ I have not experienced any symptoms of coronavirus
- ○ I am currently experiencing the symptoms but have not been diagnosed
- ○ I have already been diagnosed with coronavirus
- ○ I have been diagnosed with coronavirus and have recovered

**11. I am concerned that I will get the coronavirus.**

- ○ Strongly agree
- ○ Agree
- ○ Disagree
- ○ Strongly disagree
- ○ Prefer not to answer

**12. I am concerned that my child will get the coronavirus. (for parents only)**

- ○ Strongly agree
- ○ Agree
- ○ Disagree
- ○ Strongly disagree
- ○ Prefer not to answer

***COVID-19(coronavirus) is a new disease and there is limited information on its risk factors. However, based on currently available information, some high-risk conditions can include those in the following question:***

**13. Some behaviors and health conditions may be related to coronavirus, which we would like to understand better. Please check if any of the below apply to you or a member of your immediate family who live with you. (check all that apply)** *

| | No one in my family | Myself | One or more member(s) | Prefer not to answer |
|---|---|---|---|---|

| | | | of my family | |
|---|---|---|---|---|
| Diagnosed by a doctor as having diabetes | ◯ | ◯ | ◯ | ◯ |
| Diagnosed by a doctor with heart disease | ◯ | ◯ | ◯ | ◯ |
| Diagnosed by a doctor as having auto-immune conditions (such as celiac's disease, Crohn's disease, rheumatoid arthritis, etc.) or going through cancer treatment | ◯ | ◯ | ◯ | ◯ |
| Diagnosed by a doctor as having chronic lung disease or moderate to severe asthma. | ◯ | ◯ | ◯ | ◯ |
| Is a current smoker (cigarettes, e-cigarettes) | ◯ | ◯ | ◯ | ◯ |

**14. Which of the following actions, if any, are you currently taking to protect yourself from the coronavirus? (Check all that apply).** *

- ☐ Washing my hands with soap and water more often
- ☐ Using more disinfectants, such as hand sanitizers and cloth wipes
- ☐ Avoiding shaking hands with others
- ☐ Practicing social distancing (staying at least six feet away from other people outside of my home)
- ☐ Washing or cleaning food purchased from the grocery store.
- ☐ Other measures you are practicing? Please list

**15. How would you rate your current health status?** *

- ◯ Poor
- ◯ Fair
- ◯ Good
- ◯ Very good
- ◯ Excellent

**16. Does your family use the following?** *

| | No | Yes |
|---|---|---|
| WIC (Women Infants and Children) | ◯ | ◯ |
| SNAP Benefits / Lone Star EBT | ◯ | ◯ |
| Double Dollars Incentive Program | ◯ | ◯ |
| Medicaid/Texas Health Steps | ◯ | ◯ |
| Medicare | ◯ | ◯ |
| Free/Reduced meals at school | ◯ | ◯ |

| | | |
|---|---|---|
| CHIP (Children's Health Insurance Program) | ○ | ○ |

**17. How many people live in your home? (NUMBERS ONLY)**

| | # |
|---|---|
| Children (17 years or younger) | |
| Elders (65 years or older) | |
| Adults (18 years or older, and younger than 65 | |

**18. How do you most identify yourself? (Select only one)**

- ○ Black or African American
- ○ Mexican-American, Latino or Hispanic
- ○ White, Caucasian, or Anglo
- ○ Asian (Chinese, Indian, or another Asian country)
- ○ Native Hawaiian or Other Pacific Islander
- ○ Native American or Alaska Native
- ○ Other [specify]

**19. (Parents only) How do you most identify your child? (Select one response for only one child in your family)**

- ○ Black or African American
- ○ Mexican-American, Latino or Hispanic
- ○ White, Caucasian, or Anglo
- ○ Asian (Chinese, Indian, or another Asian country)
- ○ Native Hawaiian or Other Pacific Islander
- ○ Native American or Alaska Native
- ○ Other [specify]

**20. What language(s) do you speak most of the time at home? (Select only one)**

- ○ Most or only English
- ○ Both English and Spanish equally
- ○ Most or only Spanish

○ Other language

**21. What is YOUR gender?** *

○ Male

○ Female

**22. What is YOUR date of birth?**

**23. What is YOUR age?**

**24. What is your home zip code?**

**25. First name:** *

**26. Last Name:** *

**27. If you have a Brighter Bites keychain with 6 numbers, write the numbers here (see photo example below):**

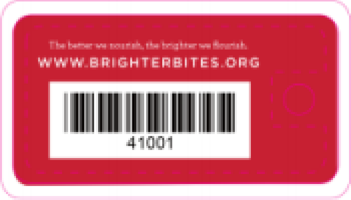


**28. Brighter Bites School Name:** *

**29. Please share your greatest concern at this time, or any other thoughts you would like to share with us.**

Thank you for completing this form. You may click next to submit.

<< Previous Next >>
