# Supplementary material for: Using a rapid assessment methodology to identify and address immediate needs among low-income households with children during COVID-19
Source: PLoS One. 2020 Oct 1;15(10):e0240009. doi: 10.1371/journal.pone.0240009 (PMC7529270; doi:10.1371/journal.pone.0240009)
Supplement: S2 File — (PDF) [file pone.0240009.s003.pdf]

COVID-19 Response Form / Formulario de Respuesta COVID-19

67% Complete

Queridos Padres,

Estamos realizando una encuesta para comprender cómo se está preparando y hacer frente a la propagación del COVID-19 (coronavirus), especialmente cuando se trata de sus necesidades con los alimentos.

- Por favor, responda lo mejor que pueda.
- No hay respuestas correctas o incorrectas.
- No existe ningún riesgo al completar esta encuesta. Al completar la encuesta, usted está aceptando participar en ella.

Brighter Bites Respuesta al COVID-19 Encuesta para Padres

1. ¿Cuánto ha visto o escuchado sobre el COVID-19 (coronavirus)? *

- ◯ Mucho
- ◯ Suficiente
- ◯ No mucho
- ◯ Nada en absoluto

2. Debido al coronavirus, ¿le preocupa alguno de los siguientes aspectos en relación con usted y su familia? (marque todo lo que corresponda) *

- ☐ Estabilidad financiera
- ☐ Mi situación financiera cambiará en un futuro cercano
- ☐ Disponibilidad de comida
- ☐ Asequibilidad de alimentos
- ☐ Disponibilidad y/o asequibilidad de vivienda
- ☐ Acceso a medios de transporte confiables
- ☐ Acceso a guarderías infantiles
- ☐ Acceso a su clínica/médico
- ☐ Otro

3. DURANTE LOS ÚLTIMOS 7 DÍAS, ¿CUÁNTAS VECES SU FAMILIA HIZO LO SIGUIENTE?: *

	Nunca	1-2 veces por semana	3-4 veces porsemana	5-6 veces porsemana	7+ veces porsemana

¿Comer alimentos de cualquier tipo de restaurante? Esto incluye restaurantes como, de comida rápida, restaurantes de servicio de comida en el sitio, restaurantes tipo buffet, tiendas de tacos, tiendas de donuts, y lugares de pizza.	○	○	○	○	○

4. Debido al coronavirus, ¿ha cambiado su frecuencia de comer en restaurantes? *

- ○ Aumentado
- ○ Disminuido
- ○ Se mantiene igual

5. ¿Qué tan cierta encuentra la siguiente declaración? Marque una opción de respuesta para cada declaración. DEBIDO AL coronavirus: *

	A menudo Verdadero	A veces Verdadero	Nunca Verdadero
Le preocupa quedarse sin comida antes de obtener dinero para poder comprar más.	○	○	○
La comida que compre no dure y no tenga dinero para comprar más.	○	○	○

6. Debido al coronavirus, actualmente ¿con qué frecuencia compra u obtiene frutas y verduras y otros comestibles para la familia en una gran tienda de comestibles o en un supermercado? (como Randall's, HEB, Kroger's, Fiesta, Whole Foods, Sprouts, Sam's club, Costco, Wal-mart o Target)) *

- ○ Nunca
- ○ Menos de una vez al mes
- ○ 1-2 veces al mes
- ○ 1 vez por semana
- ○ 2+ veces por semana

7. Debido al coronavirus, actualmente ¿con qué frecuencia compra u obtiene frutas y verduras y otros alimentos para la familia desde estos lugares? *

	Nunca	Menos de una vez al mes	1-2 veces al mes	1 vez por semana	2+ veces por semana
Una pequeña tienda local o tienda de la esquina (generalmente de un propietario local y no vende gas), o una tienda de conveniencia (como 7-11 o un mini mercado que usualmente vende gas)	○	○	○	○	○
Un mercado de agricultores/cooperativa de alimentos/puesto de granjas	○	○	○	○	○
Un banco de alimentos/despensa de alimentos, u otras distribuciones de alimentos	○	○	○	○	○

8. En este momento, ¿Cómo compran usted o los miembros de su familia en este supermercado o supermercados? (Marque todo lo que corresponda) *

- ☐ Físicamente compra dentro de la tienda
- ☐ Compra en línea y recoge en la tienda
- ☐ Compra en línea y se lo dejan en su domicilio

9. Debido al coronavirus, ¿ha cambiado su consumo de frutas y verduras? *

- ◯ Aumentado
- ◯ Disminuido
- ◯ Se mantiene igual

10. ¿Cuál de la siguiente información es cierta para usted? (Según la información de los CDC, los síntomas del coronavirus incluyen: fiebre, tos, ahogo) *

- ◯ No he experimentado ningún síntoma del coronavirus
- ◯ Actualmente estoy experimentado los síntomas pero no me han diagnosticado
- ◯ Ya me diagnosticaron con coronavirus
- ◯ Me han diagnosticado con coronavirus y me he recuperado

11. Me preocupa que pueda contagiarme con el coronavirus

- ◯ Totalmente de acuerdo
- ◯ De acuerdo
- ◯ No estoy de acuerdo
- ◯ Totalmente en desacuerdo
- ◯ Prefiero no responder

12. Me preocupa que mi hijo se contagie con el coronavirus (sólo para los padres)

- ◯ Totalmente de acuerdo
- ◯ De acuerdo
- ◯ No estoy de acuerdo
- ◯ Totalmente en desacuerdo
- ◯ Prefiero no responder

COVID-19 (coronavirus) es una enfermedad nueva y existe información limitada sobre sus factores de riesgo. Sin embargo, según la información disponible actualmente, algunas condiciones de alto riesgo pueden incluir las de las siguientes preguntas:

13. Algunos comportamientos y condiciones de salud pueden estar relacionados con coronavirus, por lo que nos gustaría entender mejor. Por favor verifique si alguno de los siguientes puntos aplica para usted o para algún miembro de su familia inmediata que viva con usted. (marque todo lo que corresponda) *

	Nadie en mi familia	Yo mismo	Uno o más de un miembro(s) de mi familia	Prefiero no responder
Diagnosticado por un médico como diabético	○	○	○	○
Diagnosticado por un médico con alguna enfermedad cardíaca	○	○	○	○
Diagnosticado por un médico por tener enfermedades autoinmunes (como la enfermedad celíaca, la enfermedad de Crohn, artritis reumatoide, etc.) o pasando por un tratamiento contra el cáncer.	○	○	○	○
Diagnosticado por un médico por tener enfermedad pulmonar crónica o asma de moderada a severa.	○	○	○	○
Es un fumador actual (cigarrillos, cigarillos electónicos)	○	○	○	○

14. ¿Cuál de las siguientes acciones, si existe alguna, está tomando actualmente para protegerse del coronavirus? (Marque todo lo que corresponda). *

- ☐ Lavando mis manos con agua y jabón con mayor frecuencia.
- ☐ Usando más desinfectantes, como desinfectantes para las manos y toallitas húmedas.
- ☐ Evitando darle la mano a otros
- ☐ Practicando el distanciamiento social (fuera de mi casa, permanecer al menos a seis pies de distancia de otras personas)
- ☐ Lavando o limpiando los alimentos comprados en el supermercado.
- ☐ Otro

15. ¿Cómo calificaría su estado de salud actual? *

- ○ Pobre
- ○ Aceptable
- ○ Bueno
- ○ Muy bueno
- ○ Excelente

16. ¿Su familia usa lo siguiente? (Seleccione sólo una respuesta por cada opción) *

	No	Sí
WIC (Mujeres, Bebés y Niños)	○	○
Beneficios SNAP / Lone Star EBT	○	○
Programa de incentivos de dólares dobles	○	○

Medicaid / Pasos de salud de Texas	◯	◯
Medicare	◯	◯
Comidas gratis / reducidas en la escuela	◯	◯
CHIP (Programa de seguro médico para niños)	◯	◯

17. ¿Cuántas personas viven en tu casa? (SOLO NUMEROS)

	#
Niños (17 años o menores)	
Ancianos (65 años o mayores)	
Adultos (18 años o mayores, y menores de 65 años)	

18. ¿Cómo te identificas mejor? (Seleccione sólo uno)

◯ Negro o Afro Americano

◯ Mejicano-Americano, Latino o Hispano

◯ Blanco, Caucásico, o Anglosajón

◯ Asiático (Chino, Hindú, o de otro país Asiático)

◯ Nativo Hawaiano o de Otra Isla del Pacífico

◯ Nativo Americano o Nativo de Alaska

◯ Otro [especifique]

19. (Sólo Padres) ¿Cómo identifica mejor a su hijo? (Seleccione una respuesta por cada hijo en su familia)

◯ Negro o Afro Americano

◯ Mejicano-Americano, Latino o Hispano

◯ Blanco, Caucásico, o Anglosajón

◯ Asiático (Chino, Hindú, o de otro país Asiático)

◯ Nativo Hawaiano o de Otra Isla del Pacífico

◯ Nativo Americano o Nativo de Alaska

◯ Otro [especifique]

20. ¿Qué idioma(s) habla la mayor parte del tiempo en casa? (Seleccione sólo uno)

◯ La mayoría o sólo inglés

◯ Ambos inglés y español por igual

◯ La mayoría o sólo español

◯ Otro idioma

21. ¿Cuál es TU género?

◯ Masculino

◯ Femenino

22. ¿Cuál es TU fecha de nacimiento?

23. ¿Cuál es TU edad en años?

24. ¿Cuál es el código postal de su casa?

25. Primer Nombre (del padre): *

26. Apellido: *

27. Si tiene usted un llavero de Brighter Bites con 6 numeros, escribe los números aquí (Vea el ejemplo de la foto a continuación):

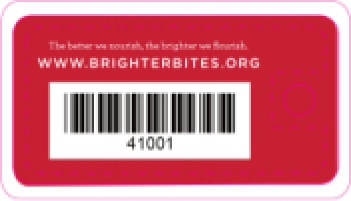

28. Escuela de BB: *

29. Comparta su mayor preocupación en este momento, o cualquier otro pensamiento que le gustaría compartir con nosotros:

Gracias por completar este formulario. Puede hacer clic en Siguiente para enviar.

<< Previous Submit
